# Association between sarcopenia and respiratory function in elderly orthopedic outpatients

**DOI:** 10.1097/MD.0000000000029365

**Published:** 2022-07-22

**Authors:** Yoshihito Tomita, Satoshi Mizukami, Takayuki Nishimura, Kazuhiko Arima, Yasuyo Abe, Mitsuo Kanagae, Kiyoshi Aoyagi

**Affiliations:** a School of Rehabilitation, Department of Physical Therapy, Tokyo Professional University of Health Sciences, Tokyo, Japan; b Department of Public Health, Nagasaki University Graduate School of Biomedical Sciences, Nagasaki, Japan; c Department of Rehabilitation, Nishi-Isahaya Hospital, Isahaya, Japan; d Department of Human Science, Faculty of Design, Kyushu University, Fukuoka, Japan; e Department of Health and Nutrition Sciences, Nishikyushu University, Kanzaki, Japan.

**Keywords:** cross-sectional study, elderly, Japanese, orthopedic outpatient, respiratory function, sarcopenia

## Abstract

The relationship between sarcopenia and respiratory function has not been investigated in elderly Japanese orthopedic outpatients. This study aimed to assess the relationship between sarcopenia and respiratory function in elderly orthopedic outpatients in Japan.

Elderly outpatients (n = 102; aged ≥65 years) with orthopedic diseases were enrolled in the study. Muscle mass was measured using the bioelectrical impedance analysis. Grip strength and walking speed were measured (2 trials). The respiratory function (e.g., percent vital capacity [VC] and percent forced expiratory volume in 1 second) was also measured. The association between sarcopenia and respiratory function was assessed using logistic regression analysis, adjusting for sex, comorbidities, and smoking status.

The mean ages for men and women were 77.7 ± 8.3 and 76.5 ± 6.8 years, respectively, and the overall prevalence of sarcopenia was 25.5% (30.0% and 24.4% in men and women, respectively). The study demonstrated that low respiratory function, which was represented by decreased percent VC, was associated with sarcopenia in outpatients visiting an orthopedic department (odds ratio, 1.73; 95% confidence interval: 1.02–2.97).

Sarcopenia was significantly associated with a lower percentage of VC in orthopedic outpatients after adjustment for sex, comorbidity, and smoking. Further studies are needed to determine the causality.

## 1. Introduction

Sarcopenia (ICD-10 code M62.84) was defined as the “age-related loss of muscle mass and function” by Rosenberg.^[1]^ The causes of sarcopenia were classified as age-related, disuse, inadequate nutrition, endocrine and neurodegenerative diseases, and cachexia.^[2]^ Sarcopenia increases the risk of negative health outcomes, such as falls, fractures, dependency, use of hospital services, institutionalization, poor quality of life, and mortality.^[3,4]^

Knee and hip pain may directly contribute to the progression of sarcopenia and increase fall risk in older women.^[5]^ Patients with orthopedic disease are likely to become inactive due to pain^[6]^; therefore, they may be at a higher risk of developing sarcopenia. The prevalence of sarcopenia is 11.5% and 16.7% in older Japanese community-dwelling men and women, respectively.^[7]^ Forty percent and 26% of patients with and without chronic low back pain, respectively, meet the criteria for sarcopenia.^[8]^ Moreover, its prevalence is 37.1% in patients with rheumatoid arthritis.^[9]^ Therefore, patient with musculoskeletal disorders are greatly affected by sarcopenia.

Diaphragm muscle mass loss was observed in aged mice.^[10]^ Sarcopenia may limit the ability of the diaphragm muscle mass to accomplish expulsive, non-ventilatory behaviors (e.g., cough) that are essential for airway clearance.^[10]^ As a result, these changes in the diaphragm muscle mass may contribute to respiratory complications with aging.^[10]^

Low muscle mass was found to be an independent risk factor for decreased pulmonary function in healthy Korean men and women aged >65 years.^[11]^ Moreover, the peak expiratory flow rate was reported to be associated with sarcopenia in Japanese community-dwelling older adults.^[12]^ However, the relationship between sarcopenia and respiratory function has not been investigated in elderly Japanese orthopedic outpatients. Thus, the purpose of this study was to assess the relationship between sarcopenia and respiratory function in elderly orthopedic outpatients in Japan.

## 2. Methods

Participants were recruited from patients with orthopedic diseases who visited the Nishi-Isahaya Hospital and enrolled voluntarily in this observational study. Written consent forms were available in Japanese to ensure a comprehensive understanding, and each participant provided written informed consent. This study was approved by the Ethics Committee for Human Use of Nagasaki University on June 20, 2016 (project registration no. 16060293).

The participants included 109 elderly outpatients aged ≥65 years with orthopedic diseases. We carried out a power analysis that showed that the sample size was sufficient for our statistical analyses (G*Power ver. 3.1: test family [χ^2^ test], statistical test [goodness of fit test: contingency tables], effect size = 0.40, alpha error = 0.05, 1-beta error = 0.80, total sample size = 81).^[13]^ G*Power was designed as a general stand-alone power analysis program for statistical tests commonly used in social and behavioral research.^[13]^ All participants had sufficient cognitive function to complete the questionnaire and were asked if they had any comorbidities (heart, lung disease, stroke, or diabetes mellitus). In addition, information on current smoking status (yes/no) was collected. Diagnosis of musculoskeletal disorders (osteoarthritis, rheumatoid arthritis, fracture and others) was collected. The sites of pain (shoulder, elbow, wrist, finger, hip, knee, ankle, foot, upper back pain, mid back pain and low back) were collected, but the severity of pain was not assessed.

Height (m) and weight (kg) were measured with participants wearing light clothing and without shoes and body mass index (BMI) was calculated as weight divided by height squared (kg/m^2^).

The participants were classified as having sarcopenia based on muscle mass, muscle strength, and physical performance. The classification was established on the recommendations of the Asian Working Group for Sarcopenia.^[14]^ These recommendations defined sarcopenia as age >60 years, a low handgrip strength (<28 kg and 18 kg in men and women, respectively) and/or slower walking speed (<1.0 m/s), low appendicular muscle mass index (AMI; <7.0 and 5.7 kg/m^2^ in men and women, respectively). Participants without low muscle mass, strength, or low physical performance were classified as normal.

Muscle mass was measured by bioelectrical impedance analysis using an InBody 430 (InBody Japan Inc., Tokyo, Japan). The bioelectrical impedance analysis method requires participants to step onto a platform and remain in the standing position for approximately 30 seconds. Appendicular skeletal muscle mass was calculated as the sum of the muscle masses of the 4 limbs. The absolute appendicular muscle mass was converted to an AMI, which was calculated by dividing the absolute appendicular muscle mass by height in meters squared (kg/m^2^).

The grip strength of the dominant hand was measured using a Jamar hydraulic hand dynamometer (Jafayette Instrument Company, Inc., Jafayette, IN). The best performance of the 2 trials was accepted as the result.

The walking speed was calculated as the time required for participants to walk a 10-m course at their usual pace (usual walking speed; average of 2 trials).

An electronic spirometer (Microspiro HI-205, Nihon-kohden Inc., Tokyo, Japan) was used to measure vital capacity (VC), VC as a percentage of predicted value (percent VC), forced vital capacity (FVC), forced expiratory volume in 1 second (FEV1), and percent FEV1 (FEV1/VC). The participants were seated comfortably in a chair. For the measurement of VC, participants were asked to breathe spontaneously several times, exhale until the point of maximal expiration, inhale to the point of maximal inspiration, and exhale again. For the measurement of FVC, they were asked to inhale to the point of maximal inspiration and exhale as quickly as possible over the following 6 seconds. The effort during the measurement was evaluated using a flow–volume curve. The VC was measured first, followed by the FVC. After several practice attempts, measurements were performed once for each participant. However, when a participant appeared to exert submaximal effort, measurements were repeated until maximal effort was achieved. The best values for each parameter were used in the analysis.

### 2.1. Statistical analysis

We used the Shapiro–Wilk test for the normality test. Comparisons of variables between the sarcopenia and the normal group were performed using Student *t* test for continuous variables or Fisher exact test for categorical variables. The association between sarcopenia and respiratory function was assessed using logistic regression analysis, adjusting for sex, comorbidities, and smoking status. The Hosmer–Lemeshow test was used to evaluate the difference between the observed and predicted prevalence in the multivariate logistic regression analysis. Odds ratios and 95% confidence intervals were calculated. Statistical significance was set at *P* < .05. All statistical analyses were performed using IBM SPSS Statistics version 27 (IBM Corp., Armonk, NY).

## 3. Results

**Figure 1.**
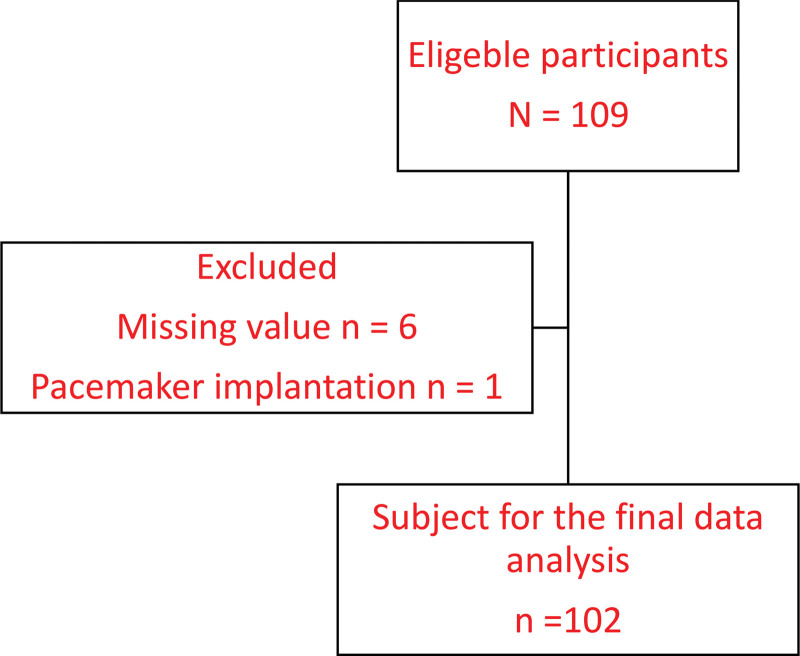
Flow diagram.

Participants with missing values (n = 6) for any variables and pacemaker implantation (n = 1) were excluded from the analysis, leaving the remnants (n = 102) for the final data analysis (Fig. 1). The prevalence of musculoskeletal disorders of the participants was osteoarthritis (n = 47, 46.1%), rheumatoid arthritis (n = 4, 3.9%), fracture (n = 44, 43.1%), and others (n = 7, 6.9%). All participants experienced at least 1 musculoskeletal pain. The sites of pain were shoulder (n = 71, 69.6%), elbow (n = 13, 12.7%), wrist (n = 12, 11.8%), finger (n = 11, 10.8%), hip (n = 17, 16.7%), knee (n = 52, 51.0%), ankle (n = 16, 15.7%), foot (n = 11, 10.8%), upper back pain (n = 24.5, 25.0%), mid back pain (n = 13, 12.7%), and low back pain (n = 67, 65.7%).

**Figure 2.**
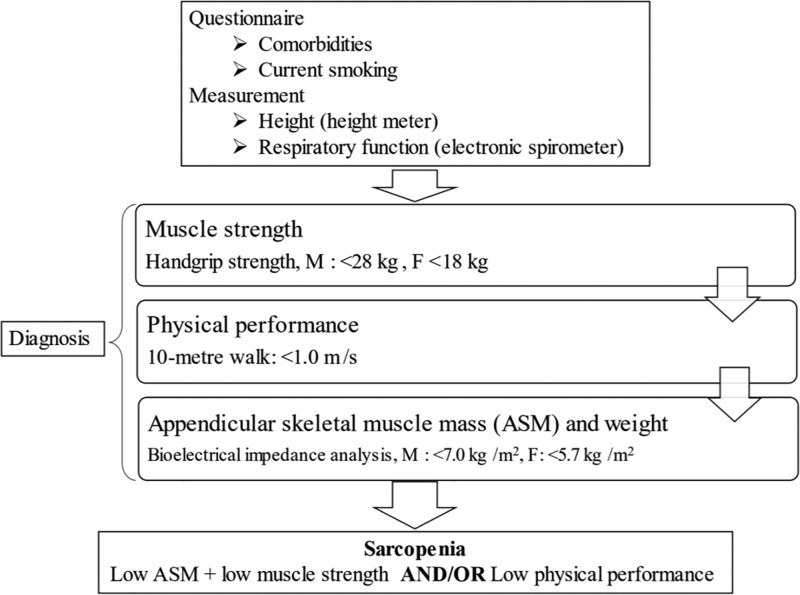
Study flow. F = female, M = male.

The study flow is shown in Figure 2. Table 1 shows the characteristics of male and female participants. The mean ages of men and women were 77.7 ± 8.3 and 76.5 ± 6.8 years, respectively. Women had significantly lower AMI, grip strength, VC, and FEV1 than men (*P* < .001). The mean percent VC was greater in women than in men (*P* = .016). The number of participants who were current smokers was higher among men (*P* < .001).

**Table 1:** Characteristics of the participants (N = 102).

Variable	All (N = 102)		Men (n = 20)		Women (n = 82)		*P* value
	Mean	SD	Mean	SD	Mean	SD	
Age, y	76.8	7.1	77.7	8.3	76.5	6.8	.512
Body mass index, kg/m^2^	23.5	3.8	22.5	2.9	23.8	4.0	.175
AMI, kg/m^2^	6.1	0.9	7.0	0.7	5.9	0.8	<.001*
Grip strength, kg	22.0	6.4	29.9	7.5	20.0	4.4	<.001*
Walking speed, m/s	1.1	0.2	1.1	0.2	1.1	0.2	.398
VC, L	2.5	0.6	3.0	0.7	2.3	0.4	.001*
Percent VC, %	107.0	20.8	97.0	19.3	109.4	20.6	.016*
FEV1, L	1.77	0.4	2.1	0.5	1.7	0.4	.001*
Percent FEV1, %	72.0	9.1	71.6	10.6	72.0	8.7	.879

	Number	%	Number	%	Number	%	
Comorbidity,† yes	78	76.5	12	60.0	66	84.6	.076
Heart disease, yes	17	16.7	3	15.0	14	17.1	.823
Lung disease, yes	14	13.7	5	25.0	9	11.0	.102
Stroke, yes	7	6.9	2	10.0	5	6.1	.536
Diabetes mellitus, yes	11	10.8	2	10.0	9	11.0	.900
Smoking, yes	19	18.6	15	75.0	4	4.9	<.001*

Student *t* test for continuous variables; Fisher exact test for categorical variables. Men vs women.

AMI = appendicular muscle mass index, FEV1 = forced expiratory volume in 1 second, SD = standard deviation, VC = vital capacity.

* *P* value <.05.

† Heart, lung disease, stroke, or diabetes mellitus.

The overall prevalence of sarcopenia in the present study was 25.5% (30.0% and 24.4% in men and women, respectively; Table 2).

**Table 2:** Prevalence of sarcopenia (N = 102).

	All (N = 102)		Men (n = 20)		Women (n = 82)	
	Number	%	Number	%	Number	%
Low AMI, kg/m^2^	42	41.2	7	35.0	35	42.7
Low walking speed	38	37.3	7	35.0	31	37.8
Low grip strength	29	28.4	7	35.0	22	26.8
Sarcopenia	26	25.5	6	30.0	20	24.4

AMI = appendicular muscle mass index.

Table 3 shows the comparison of variables between the sarcopenia and normal groups. In all participants, the sarcopenia group had lower BMI, AMI, grip strength, usual walking speed, VC, percent VC, and FEV1 (all *P* < .05), whereas percent FEV1, comorbidity, and smoking did not differ significantly. Men and women with sarcopenia had significantly lower BMI (*P* = .002 and *P* < .001 in men and women, respectively), AMI (*P* = .006 and *P* < .001 in men and women, respectively), grip strength (*P* = .003 and *P* < .001 in men and women, respectively), VC (*P* = .005 and *P* = .013 in men and women, respectively), and FEV1 (*P* = .037 and *P* = .021 in men and women, respectively) compared to normal. Higher age (*P* = .032) and lower percent VC (*P* = .014) were associated with sarcopenia in men. Women with sarcopenia had significantly slower walking speed (*P* < .001).

**Table 3:** Comparison of variables between the sarcopenia and the normal group (N = 102).

Variable		Sarcopenia	Normal	*P* value
All	Age, y	78.9 ± 8.0	76.1 ± 6.6	.082
	Body mass index, kg/m^2^	21.2 ± 2.4	24.3 ± 3.9	<.001*
	AMI, kg/m^2^	5.4 ± 0.6	6.4 ± 0.9	<.001*
	Grip strength, kg	18.6 ± 4.6	24.0 ± 6.5	<.001*
	Walking speed, m/s	0.9 ± 0.2	1.1 ± 0.2	<.001*
	VC, L	2.2 ± 0.4	2.6 ± 0.6	.003*
	Percent VC, %	98.3 ± 19.1	109.9 ± 20.7	.014*
	FEV1, L	1.6 ± 0.4	1.8 ± 0.4	.013*
	Percent FEV1, %	72.3 ± 8.9	71.8 ± 9.2	.802
	Comorbidity,† yes	22 (84.6)	56 (73.7)	.257
	Smoking, yes	6 (23.1)	13 (17.1)	.500
Men	Age, y	83.7 ± 8.5	75.1 ± 7.1	.032*
	Body mass index, kg/m^2^	19.7 ± 3.0	23.7 ± 1.9	.002*
	AMI, kg/m^2^	6.2 ± 0.6	7.3 ± 0.3	.006*
	Grip strength, kg	23.5 ± 5.5	33.7 ± 6.2	.003*
	Walking speed, m/s	1.0 ± 0.2	1.1 ± 0.2	.334
	VC, L	2.4 ± 0.4	3.2 ± 0.6	.005*
	Percent VC, %	81.6 ± 16.6	103.6 ± 16.7	.014*
	FEV1, L	1.8 ± 0.3	2.2 ± 0.5	.037*
	Percent FEV1, %	77.5 ± 10.2	69.1 ± 10.0	.913
	Comorbidity,† yes	4 (66.7)	8 (57.1)	.545
	Smoking, yes	5 (83.3)	10 (71.4)	.517
Women	Age, y	77.4 ± 7.4	76.3 ± 6.6	.515
	Body mass index, kg/m^2^	21.6 ± 2.1	24.5 ± 4.2	<.001*
	AMI, kg/m^2^	5.2 ± 0.4	6.2 ± 0.8	<.001*
	Grip strength, kg	17.1 ± 3.1	21.8 ± 4.2	<.001*
	Walking speed, m/s	0.9 ± 0.2	1.2 ± 0.2	<.001*
	VC, L	2.1 ± 0.4	2.4 ± 0.4	.013*
	Percent VC, %	103.3 ± 17.1	111.3 ± 21.4	.133
	FEV1, L	1.5 ± 0.4	1.7 ± 0.3	.021*
	Percent FEV1, %	70.8 ± 8.1	72.4 ± 9.0	.474
	Comorbidity,† yes	18 (90)	48 (77.4)	.184
	Smoking, yes	1 (5)	3 (4.8)	.681

Mean ± standard deviation, number (%). Student *t* test for continuous variables; Fisher exact test for categorical variables.

AMI = appendicular muscle mass index, FEV1 = forced expiratory volume in 1 second, VC = vital capacity.

* *P* value <.05.

† Heart, lung disease, stroke, or diabetes mellitus.

After adjustment for sex, comorbidity, and smoking, the sarcopenia group had a significantly lower percentage of VC in orthopedic outpatients (Table 4). The percent FEV1 was not significantly associated with sarcopenia.

**Table 4:** Association between sarcopenia and respiratory function (N = 102).

Variables	Unit	Odds ratio (95% confidence interval)
Percent VC	−20.8%*	1.73 (1.02–2.97)†
Adjusted factors		
Sex	Women	1.07 (0.20–5.71)
Comorbidity‡	Yes	1.87 (0.53–6.55)
Smoking	Yes	0.78 (0.15–4.19)
Percent FEV1	−9.1%*	0.96 (0.61–1.53)
Adjusted factors		
Sex	Women	0.83 (0.18–3.97)
Comorbidity‡	Yes	2.15 (0.64–7.25)
Smoking	Yes	0.69 (0.15–3.27)

Logistic regression analysis.

FEV1 = forced expiratory volume in 1 second, VC = vital capacity.

* 1 standard deviation.

† *P* value <.05.

‡ Heart, lung disease, stroke, or diabetes mellitus.

## 4. Discussion

### 4.1. Prevalence of sarcopenia

In the present study, the prevalence of sarcopenia was 30.0% and 24.4% in men and women, respectively, a higher rate than that reported in previous reports of community-dwelling elderly persons.^[15–17]^ In addition, all the participants experienced at least 1 musculoskeletal pain. Pain was a significant predictor of sarcopenia transition over a 9-year period,^[18]^ which may reflect avoidance of physical activity due to fear of pain.^[6]^ Sarcopenia is suspected to be a serious problem among elderly orthopedic outpatients.

### 4.2. Sarcopenia and low pulmonary function

We demonstrate that low pulmonary function, after adjustment for sex, comorbidity, and smoking, represented by decreased percent VC, is associated with sarcopenia based on the consensus of the Asian Working Group for Sarcopenia^[14]^ in outpatients visiting an orthopedics department. Respiratory muscles, especially inspiratory ones, are significantly related to limb muscle strength and skeletal muscle mass.^[19]^ As aging progresses, skeletal muscle mass decreases, and respiratory muscle mass may also decrease,^[11]^ which is consistent with our results.

### 4.3. Sarcopenia and percent FEV1

In the present investigation, percent FEV1 (FEV1/VC) was not associated with sarcopenia, unlike FEV1 and VC, and these findings are consistent with previous ones.^[11,20]^ VC usually represents lung volume, and FEV1, the expiratory flow rate; therefore, VC and FEV1 can be reduced in participants with low muscle mass, because they may have weakened abilities to inflate and deflate their lungs.^[21]^ Here, both VC and FEV1 were lower in the sarcopenia than in the normal group. However, percent FEV1 (FEV1/VC) represents upper airway obstruction and may remain invariant, regardless of muscle mass,^[20,22]^ which may in part be the reason for the lack of association between percent FEV1 and sarcopenia.

### 4.4. Sarcopenia, comorbidity, and smoking

The prevalence of sarcopenia is increased in patients with chronic heart failure.^[23]^ Hemiparetic stroke leads to muscle abnormalities with a combination of denervation, disuse, remodeling, and spasticity, which may account for a complex pattern of phenotype shift and atrophy.^[24,25]^ Muscle strength loss is associated with diabetes and smoking.^[26]^ Thus, a logistic regression analysis adjusting for these variables was performed.

### 4.5. Limitations

The present study had several limitations. First, because the study was cross-sectional in nature, causal relationships between sarcopenia and low respiratory function were not possible. Hence, a longitudinal study is required to determine the causality. Second, the findings were obtained from elderly Japanese orthopedic outpatients. Therefore, the results cannot be extrapolated to other ethnicities. Third, patients may have difficulty judging the severity of comorbidities, consequently, the severity was not evaluated. Moreover, we did not collect information regarding the medications and the severity of pain. Thus, these limitations might have contributed to the underestimation of the associations. Finally, there was a possibility of selection bias due to the single-hospital study.

## 5. Conclusions

The overall prevalence of sarcopenia in the present study was 25.5% (30.0% and 24.4% in men and women, respectively), a higher rate than that reported in previous studies of community-dwelling elderly persons. Sarcopenia was significantly associated with a lower percentage of VC in orthopedic outpatients after adjustment for sex, comorbidity, and smoking. Pulmonary function tests, which are regularly measured in the hospital and before surgery, may be useful predictors of sarcopenia.

## Acknowledgments

The authors thank all the participants. The authors wish to express their gratitude to the medical staff and colleagues at Nishi-Isahaya Hospital for supporting this study through their kind cooperation.

## Author contributions

YT, SM, KAr, TN, YA, MK, and KAo designed the study. YT collected and analyzed the data and drafted the manuscript. KAo supervised and assisted with data collection and analysis, and manuscript preparation. YT, SM, KAr, TN, YA, MK, and KAo advised on the study design and data analysis and edited the manuscript. All authors read and approved the final manuscript.
